# Patterns of suspected wheat-related allergy: a retrospective single-centre case note review in 156 patients

**DOI:** 10.1186/2045-7022-4-39

**Published:** 2014-11-21

**Authors:** Morten J Christensen, Esben Eller, Charlotte G Mortz, Carsten Bindslev-Jensen

**Affiliations:** Department of Dermatology and Allergy Centre, Odense Research Center for Anaphylaxis (ORCA), Odense University Hospital, Odense C, 5000 Odense, Denmark

**Keywords:** Age dependency, Natural course of wheat allergy, Wheat allergy, Wheat challenge, ω-5 gliadin

## Abstract

**Background:**

Allergy to wheat can present clinically in different forms: Sensitization to ingested wheat via the gastrointestinal tract can cause traditional food allergy or in combination with exercise, Wheat-Dependent Exercise-Induced Anaphylaxis (WDEIA). Sensitization to inhaled wheat flour may lead to occupational rhinitis and/or asthma.

**Methods:**

We retrospectively reviewed the case notes of 156 patients (age 0.7 – 73.3 years) with a case history of wheat allergy. The population was divided into three groups, 1: Wheat allergy elicited by ingestion, 2: By inhalation and 3: WDEIA. All patients were examined with detailed case history, specific IgE (sIgE), Skin Prick Test (SPT) and wheat challenge (nasal or oral ± exercise). Details of the case history were extracted from the patients´ case records.

**Results:**

Group 1: Twenty one of 95 patients were challenge positive (15 children, 6 adults). All children had atopic dermatitis, and most (13/15) outgrew their wheat allergy. Most children (13/15) had other food allergies. Challenge positive patients showed significantly higher levels of sIgE to wheat and significantly more were SPT positive than challenge negative.

Group 2: Eleven out of 13 adults with occupational asthma or rhinitis were challenge positive. None outgrew their allergy. Seven had positive sIgE and 10 had positive SPT to wheat.

Group 3: Ten of 48 (adolescent/adults) were positive when challenged during exercise. Challenge positive patients showed significantly higher levels of sIgE to ω-5-gliadin. The natural course is presently unknown.

**Conclusion:**

Wheat allergy can manifest in different disease entities, rendering a detailed case history and challenge mandatory. Patient age, occupation, concomitant allergies (food or inhalant) and atopic dermatitis are important factors for evaluation.

## Introduction

Wheat proteins can be classified into the albumin and globulin (water/salt-soluble) fraction and the gliadin and glutenin (alcohol and acid/alkali-soluble) fraction (gluten) [1]. Both water/salt-soluble and insoluble proteins have been implicated with in wheat hypersensitivity [2]. Wheat allergy, as a specific immunoglobulin-E (sIgE)-mediated reaction to wheat protein, is a complex disease due to the many allergenic components (water soluble and –insoluble) in wheat [2, 3]. Depending on the route of exposure and underlying immunological mechanisms, wheat allergy may manifest clinically in different forms: Sensitization to ingested wheat can cause traditional food allergy and in combination with exercise, Wheat-Dependent Exercise-Induced Anaphylaxis (WDEIA) [3], whereas inhalation of wheat flour can lead to occupational rhinitis and/or asthma [4–6].

Since data on wheat allergy are sparse, the aim of this study was to describe characteristics and clinical outcomes of 156 patients evaluated for a wheat related allergy.

## Methods

### Subjects

**Figure 1:**
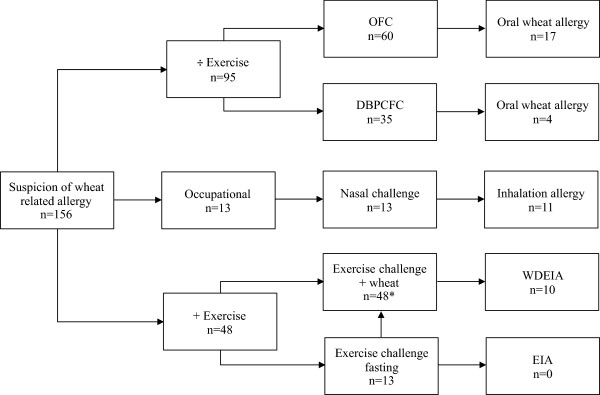
**Flowchart of patients with suspected wheat related allergy.** OFC: Open food challenge. DBPCFC: Double-blind, placebo-controlled food challenge. WDEIA: Wheat-Dependent Exercise-Induced Anaphylaxis. EIA: Exercise Inducecd Anaphylaxis. *7 patients were also challenged with aspirin and/or alcohol.

From May 2001 to September 2013, we investigated 156 patients (72 female, 84 male, age 0.7 – 73.4 years) with a case history of a type 1 immediate reaction related to wheat ingestion as part of routine clinical care. All data were collected retrospectively and anonymously from medical records by the patients´ responsible clinicians. The population was divided into three groups (Figure 1):

1. Case history of an allergic reaction to ingestion of wheat (group 1, n = 95)
2. Case history of an allergic reaction to inhalation of wheat flour (group 2, n = 13).
3. Case history of an allergic reaction to ingestion of wheat in combination with physical exercise (group 3, n = 48)

At the time of consultation in the clinic, all patients had a detailed case history recorded, and underwent clinical examination, measurement of serum sIgE, Skin Prick Test (SPT) followed by wheat challenge (nasal or oral) as part of routine clinical practice by medical staff involved in their care. If judged relevant based on case history and test results, wheat challenge was also performed during physical exercise.

### Serology and skin prick test

For measurements of serum levels of sIgE to wheat (f4) and grass (g6) were analysed before challenge (ImmunoCAP system (ThermoFischer, Uppsala, Sweden). In group 3 (WDEIA) sIgE to ω-5-gliadin (f416) was also measured. Positive results were defined as ≥0.35 kU/l.

SPT with raw wheat, rye, oat and barley together with standard inhalant panel (ALK-Abello, Copenhagen, Denmark) was performed on the forearm of the patient. A positive SPT was defined as a wheal size of ≥3 mm larger than the negative control. Histamine dihydrochloride (10 mg/ml, ALK, Copenhagen, Denmark) and physiological saline were used as positive and negative controls. Antihistamines and other drugs interfering with SPT were discontinued at least three days prior to testing. Specific IgE and SPT were performed less than one year prior to challenge.

### Challenge procedure

The challenge procedure was according to the daily routine in the Allergy Centre, including termination of interfering drugs three days prior to challenge. Informed consent was obtained verbally from all patients or – in case of minors - from parents.

A positive challenge was defined according to Sampson’s grading scale for anaphylaxis [7].

### Oral wheat challenge

To determine the clinical threshold to wheat an open food challenge (OFC) or double-blind, placebo-controlled food challenge (DBPCFC) was performed. Material for DBPCFC was produced by masking 25/50 gram wheat or placebo in a chocolate bar weighing in total 155–175 gram [8]. Increasing doses of 0.5, 1, 4, 8, 16, 32 and 95–115 gram bar were given with 30 minutes interval. In OFC increasing doses of 0.25, 0.5, 1, 2, 4, 8 and 9.25 gram to a cumulative dose of 25 gram were administered at 30 minutes interval. In selected cases and based on case-history the cumulative dose was increased up to 125 gram. The individual doses of wheat flour were mixed with, for example, yoghurt. Challenge was terminated when the patient developed signs ≥ grade 2 [7], relevant treatment was instituted and the patient kept under strict surveillance according to the Centre’s guidelines.

### Nasal challenge

Thirty mg of placebo flour (potato or maize) was administrated single blinded via a spatula and sniffed into one nostril. After 10 minutes, the procedure was repeated in the opposite nostril with 30 mg of the (most often) raw wheat. A negative outcome was repeated up to 3 times for verification. Symptoms and signs monitored during challenge were itching, sneezing, watery secretion and drop in FEV1.

### Exercise and food challenge

OFC plus exercise included a combination of ingestion of a wheat containing food with physical exercise on a treadmill (Technogym Excite + Run Now 900). As standard, the patients were given 3 slices of commercially available wheat toast bread, equivalent to 44 grams of wheat flour The wheat product was ingested 30 minutes before the exercise challenge, (in selected patients extended up to 180 minutes). Aspirin (500 mg) and/or alcohol (serum level 0.5^0^/00) were added in 5 patients and the challenge repeated on a separate day.

The exercise challenge protocol was modified from the protocol developed by Bruce et al. [9]. Challenges started with a 20–30 minute aerobic phase adapted to individual physical ability. Thereafter, based on the aerobic workload, a multistage exercise test was performed, where both pace and inclination were increased every minute to physical fatigue. Each multistage intensity cycle was followed by one minute of light workload (5 km/h and no inclination). Serum lactate was measured (Lactate Pro, Arkray, Kyoto, Japan) after the multistage exercise challenge at physical fatigue.

### Statistics

Statistical analyses were performed using statistical software (STATA version 11.1, StataCorp, College Station, TX, USA). For the purpose of statistical analysis, sIgE values <0.35 were assigned a value of zero. Statistical significance was calculated using the t-test and Fischer´s exact test and defined for all comparisons by p values <0.05. Sensitivity and specificity of sIgE to wheat for patients with a respective wheat related allergy were examined by receiver-operator characteristics (ROC)-curves [10].

## Results

Characteristics of the study population (n = 156) and results from the challenges (42 positive) are presented in Table 1. All positive challenges included objective signs (Table 1), most were grade 2 (81%), grade 3 (14%) and grade 4 (5%) according to Sampson [7] and all placebo challenges were negative (n = 35).

**Table 1:** **Challenge data and patients characteristics**

**Group 1**	**Oral wheat allergy**						
Patient no.	Age (y)	Sex	Atopic diseases	Symptoms at challenge	Other food allergies	Threshold (g)	Outgrown
1	0.7	M	AD, AB	U, E	Rye	0.75	Ch + An
2	0.7	M	AD, AB	U, D, V		20.0	An
3	0.7	M	AD	U	Egg, Milk, Rye	8.84	Ch + An
4	0.8	M	AD, AB	U, A, C, E, P	Cashew, Egg, Pistacio	15.75	An
5	0.8	F	AD, AR, AB	U, V	Egg	7.75	Ch + An
6	0.9	M	AD, AB	U	Egg	25.0	Ch + An
7	0.9	M	AD, AR	U, R, C, V	Egg, Milk, Rye	7.75	Ch + An
8	1.4	M	AD	U	Egg	25.0	Ch + An
9	1.5	M	AD	U, AB, V	Rye	4.0	No
10	2.1	M	AD, AR, AB	U, C, D, E, R	Egg, Milk	15.75	Ch + An
11	2.5	F	AD	U, E, OAS	Egg, Milk, Poppy seeds	0.25	Ch + An
12	2.7	M	AD, AB	U	Egg, Hazelnut, Peanut	25.0	An
13	3.0	M	AD, AR, AB	U	Egg	7.75	No
14	3.6	M	AD, AR, AB	U, P	Melon, Milk, Rye	8.0	Ch + An
15	5.6	M	AD	U		25.0	Ch + An
Negative 16-48	Age 0–6, F = 7, M = 26		AD = 27, AR = 5, AB = 5		Egg = 15, Milk =6, Peanut = 6, Hazelnut =3 Soya = 1, Lamb =1, Shrimp = 1, None = 1		
Negative 49-59	Age 7–18, F = 5, M = 6		AD = 6, AR = 7, AB = 7		Egg = 3, Milk = 2, Peanut = 1, None = 8		
60	33.5	F		U, C, R		36.0	No
61	37.6	F	AR	U, E		3.91	No
62	38.5	M		U, OAS, P	Rye	50.0	No
63	41.7	F	AR	U, OAS, P		50.0	No
64	52.2	F	AR	OAS, P		50.0	No
65	73.4	F	AR	U, AB, C, R		6.0	No
Negative 66-95	Age 19–67.4, F = 23, M = 7		AD = 4, AR = 11, AB = 7		Egg = 1, Plaice = 1, Sesame = 1, None = 27		
**Group 2**	**Inhalation wheat allergy**						
Patient no.	Age (y)	Sex	Atopic diseases	Symptoms at challenge	Other inhalant allergies	Occupation	Outgrown
96	23.0	F		R, AB		Baker	No
97	25.4	F		R, C	Durum, Rye	Baker	No
98	29.5	F		R		Chef	No
99	29.9	M		R, C		Baker	No
100	24.0	M	AR	R, C	Rye	Baker	No
101	25.7	F	AD, AR	R, AB		Baker	No
102^	37.3	F	AR	R		Grahpic designer	No
103^	40.2	M		R	Rye	Pizza baker	-
104	41.1	F		R, C		Chef	-
105	41.6	M		R, C		Pizza baker	No
106	43.5	F	AR	R, C		Baker	No
107*	17.6	F			Spelt	Student	No
108*	33.8	M			Durum, Rye	Baker	No
**Group 3**	**Wheat dependent exercise induced anaphylaxis**						
Patient no.	Age (y)	Sex	Atopic diseases	Symptoms at challenge	sIgE to ω-5 gliadin (kUI/l)	Challenge dose (g)	Serum lactate (mM)
109	23.0	M		U	14.9	44.0	11.6
110	27.1	F	AD	U	11.5	44.0	2.3
111	27.4	F	AB	U	< 0.35	44.0	5.9
112	29.7	F		U, A	< 0.35	44.0	2.3
113	32.4	F		U	< 0.35	44.0	12.6
114	38.1	F		U	29.4	14.7	3.4
115	41.7	M		U	36.6	44.0	3.1
116	42.2	M		U	11.0	44.0	-
117	51.2	M		U	22.7	44.0	4.8
118	67.2	M		U	8.2	29.3	-
Negative 119-123	Age 7–18.9, F = 2, M = 3		AD = 1, AR = 0, AB = 1		n = 0/5 (0%)	mean 49.8 [44.0-73.0]	mean 10.0 [7.1-12.9]
Negative 124-156	Age 19–63.0, F = 14, M = 19		AD = 2, AR = 9, AB = 1		n = 20/33 (61%) mean 6.7 [0–46.7]	mean 45.4 [14.3-58.7]	mean 11.5 [4.0-18.7]

F Female, M Male, AD Atopic Dermatitis, AR Allergic Rhinitis, AB Bronchial Asthma, U Urticaria, E Erythema, D Diarrhoea, V Vomiting, A Angioedema, C Conjunctivitis, P Pruritus, R Rhinitis, AB Asthma, OAS Oral Allergy Syndrome, Ch Challenge verified, An Anamnestically verified.

^*^Negative wheat inhalation challenge.

^^^Overlapped with a negative oral wheat challenge.

### Oral wheat allergy (Group 1)

**Figure 2:**
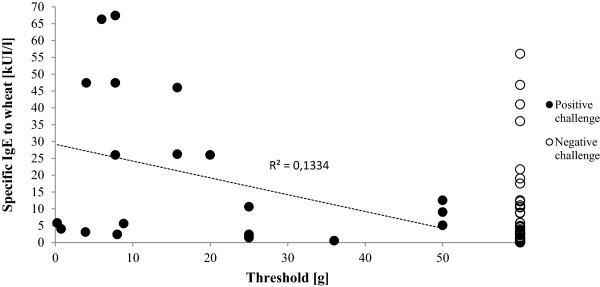
**Oral wheat challenge; comparison of specific IgE to wheat and threshold.**

**Figure 3:**
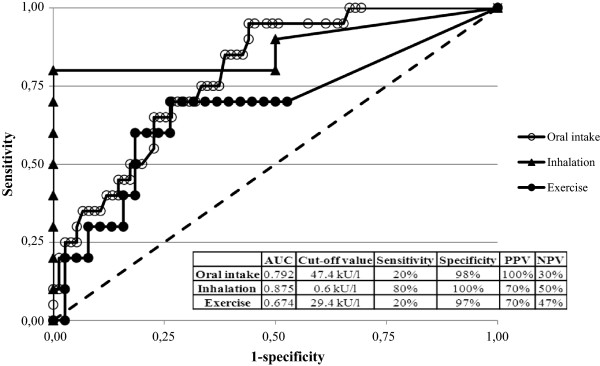
**ROC-curves of specific IgE to wheat (oral intake and inhalation) and specific IgE to ω-5 gliadin (exercise).** AUC: Area under the curve, PPV: Positive predictive value, NPV: Negative predictive value. The calculation in the "inhalation" group is only based on two negative challenges.

Twenty-one of the 95 who performed oral wheat challenges were positive; 15 of 48 were in children below 6 years of age (31%). Children (both challenge positive and negative) had significantly (p < 0.01) more often atopic diseases, especially atopic dermatitis, than adults (96% vs. 42%), Table 1. Ninety-four percent of all children and 87% of wheat challenge positive children were allergic (proven by challenge) to a least one other food. In adults only 11% had another challenge proven food allergy (p < 0.01). The clinical threshold for elicitation of objective signs varied between 25 mg and 50 g (Figure 2). We found no correlation between age of the patient and threshold and only a weak correlation between level of sIgE and threshold (r = 0.13). According to the ROC-curve analysis (Figure 3), the cut off value for sIgE to wheat producing the best sensitivity (20%) and specificity (98%) was 47.4 kU/l.

**Figure 4:**
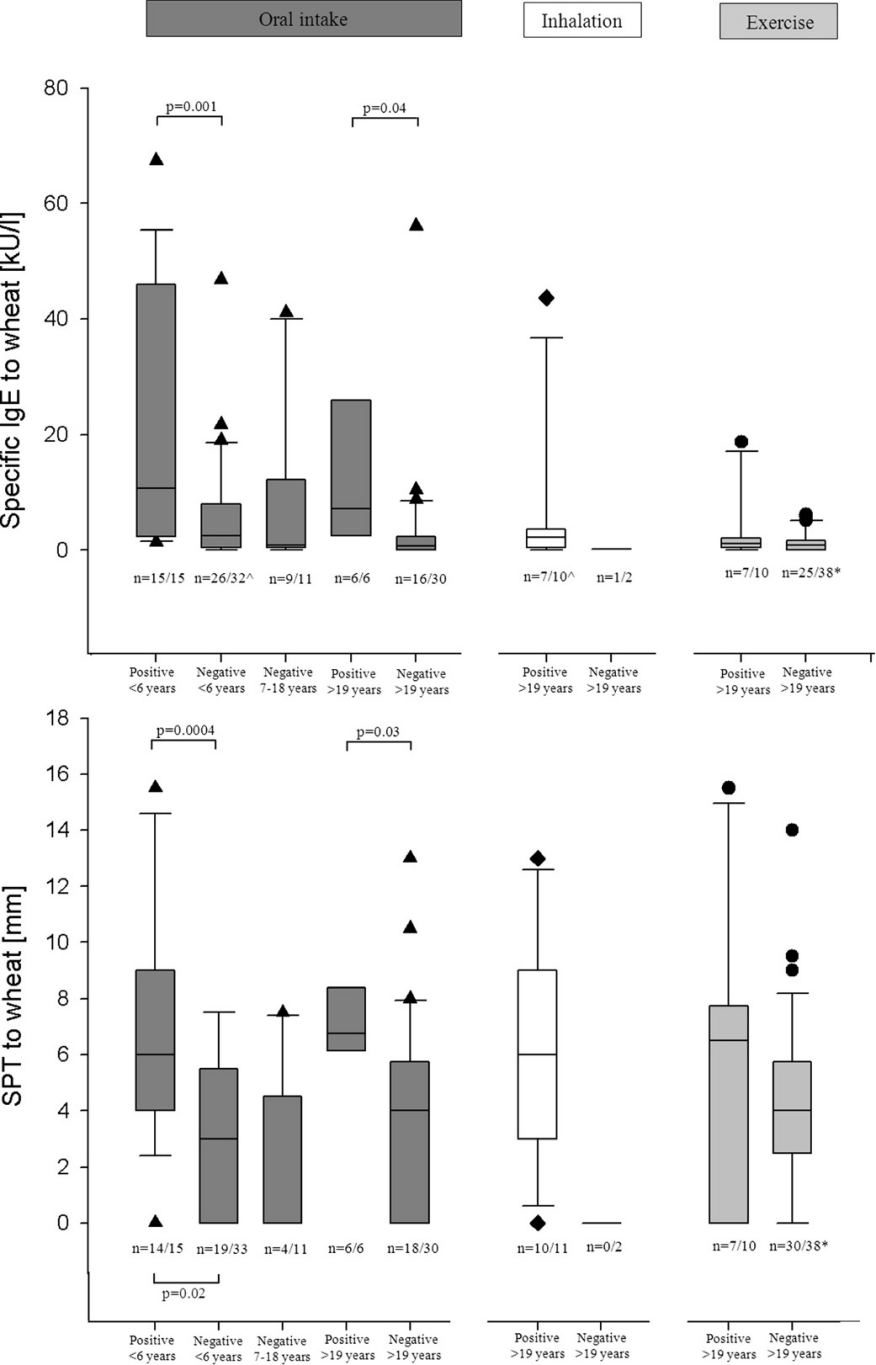
**Comparison of specific IgE and SPT to wheat in the respective groups.** *5 patient age 12–18 years included in the group, all with a negative sIgE to wheat. ^^^1 patient had no measurement of sIgE to wheat.

Challenge positive patients showed significantly higher levels of sIgE and larger SPT wheals to wheat, than the challenge negative group, (Figure 4). We found no significant difference between challenge positive and negative children aged 0–6 years and sensitization to grass pollen. In the challenge negative group aged 7–18 years, 9 of 11 (82%) (mean 36.6, range [0–80.0 kU/l]) were sensitized to grass. No significant differences were found among the adults.

In order to monitor the natural course of their allergy (persistence or development of tolerance) fourteen patients underwent more than one challenge: All children except two outgrew their wheat allergy (mean age 3.6 years). The median time between diagnosis and acquisition of tolerance was 23 months. Based upon telephone interview all adults had a persistent wheat allergy, none were re-challenged.

### Inhalation allergy (Group 2)

All patients tolerated ingestion of cereal foods and no patient had a history of oral wheat allergy in childhood. Four patients reacted both to wheat and to rye or durum.

All challenge positive patients developed itching, serial sneezes and rhinorrhoea within 10 minutes after challenge with wheat. Two patients (No. 96 and No. 101) developed dyspnoea and a significant decrease (≥20%) in FEV1. No significant signs were elicited during placebo challenge.

Specific IgE to grass was detected in 4 challenge positive patients (mean 4.7 range [0–29.2 kU/l] none had symptoms during the grass pollen season.

According to the ROC-curve analysis (Figure 3), the cut off value for sIgE to wheat, producing the best sensitivity (80%) and specificity (100%) was 0.6 kU/l, but this calculation is based on two patients in the negative group only.

If re-exposed to wheat flour, all challenge positive patients experienced symptoms of rhinitis, conjunctivitis or asthma. All 13 patients still tolerated ingestion of cereal foods when interviewed 1–11 years later and 5/11 patients had changed occupation.

### WDEIA (Group 3)

All 48 patients had a convincing case-history related to wheat ingestion combined with physical exercise, but only ten were positive in challenge. No patient had a history of oral wheat allergy or symptoms in response to wheat ingestion alone. Diagnosis of cholinergic urticaria or exercise-induced anaphylaxis (EIA) were excluded either by a fasting exercise challenge (n = 13) or by the patient case history (n = 35).

Three patients were challenge negative to wheat and exercise, but developed a reaction during challenge with either rye (No. 131 and 135) or oats (No.143). No patient experienced hypotension, collapse or shock during or after the exercise challenge, nor were any late reactions reported by patients.

Based on case history, in 4 patients with a negative challenge outcome, a combination of wheat and aspirin (500–1000 mg and in one of the patients also alcohol at a serum level of 0.5 ^o^/oo) was administrated 1 to 2 hours prior to challenge, again with a negative outcome of the challenge. Although the challenge positive patients had a significantly higher mean value of sIgE to ω-5-gliadin (13.4 kU/l compared to 5.8 kU/l (p < 0.05) in the negative group), the highest measured level was found in a challenge negative patient (No.129). Elevated levels of sIgE to rye and barley were found significantly more often (p < 0.05) among the challenge positive than negative patients.

According to the ROC-curve analysis (Figure 3), the cut off value for sIgE to ω-5-gliadin, producing the best sensitivity (20%) and specificity (97%), was 29.4 kU/l. Frequency of sensitization to grass showed no significant difference between the groups. SPT to wheat flour showed no difference between the positive and negative patients, (Figure 4).

Patients with a positive challenge reacted before a rise in serum lactate could be detected and thus ended up with a significantly (p < 0.01) lower serum lactate compared to the negative group.

## Discussion

Wheat allergy is a result of sensitization to wheat proteins with different characteristics (water or alcohol solubility, heat resistance) and presents with different clinical phenotypes (urticaria, rhinitis or anaphylaxis), depending on age of patient, concomitant other food or inhalant allergies, atopic dermatitis, route of exposure, sensitization pattern and cofactors such as exercise. A detailed case history and most often a food challenge is therefore mandatory in each patient. The separate disease entities have been published from different centers in detail previously [11–17], but this is the first case series describing differences and similarities between the groups from a single clinical setting.

*Age*: Children and infants present with symptoms upon oral intake of heat-treated wheat products and without involvement of cofactors, although single cases of exercise induced wheat allergy have been described [18]. Adults, on the other hand, can present with symptoms upon oral intake, by inhalation of raw wheat proteins especially in the occupational setting and after ingestion in combination with cofactors such as physical exercise.

*Patient characteristics*: The infants and children below the age of 6 years most often present with atopic dermatitis and another challenge proven food allergy, most often to hen’s egg (Table 1). Adults with a challenge proven oral wheat allergy characteristically suffer from allergic rhinitis to pollen and also describe oral itching (OAS) upon wheat ingestion (Table 1). Patients with challenge proven reaction to inhaled wheat flour, or who react to ingestion of wheat in combination with physical exercise, are rarely atopic but present with typical case histories. Unfortunately, the patients with negative challenges in all groups present with identical case histories, symptoms and signs with almost the same frequencies except OAS, rendering patient characteristics of little importance for discrimination. The vast majority (87%) of children with oral wheat allergy develop tolerance early in life whereas older children [19, 20] and adults [21] (independent of type of wheat allergy) rarely develop tolerance. In a study by Keet et al. [19], 65% acquired tolerance with a median age of 6.5 years.

*Symptoms and signs during challenge*: Oral intake of baked wheat almost exclusively elicited generalized acute urticaria in children and adults [13, 20]. In combination with exercise, however, anaphylaxis can be elicited. As expected, inhalation of raw wheat proteins elicit respiratory symptoms, most of sneezing, but occasionally also asthma [14].

*Clinical thresholds*: Patients with reactions to inhaled wheat had significantly lower thresholds than the majority of patients reacting to ingested, processed wheat. The thresholds established in group 1 (oral wheat allergy) are in accordance with previously published data in children and adults [13]. An explanation for the many challenge negative patients in group 3 (WDEIA) could be, that clinical thresholds for the majority of patients may be higher than 44 g of wheat, since we did not find any significant differences between the challenge positive and negative groups concerning case history, levels of sIgE or SPT. The influence of other co-factors such as drugs, alcohol, current infection, temperature, stress, hormonal changes as well as the concept of summation anaphylaxis diminishing the clinical threshold needs further investigation [22]. An interesting and as yet unpublished finding in group 3 (WDEIA) is that a clinical reaction take place under aerobic conditions during exercise as a significant (p < 0.01) lower serum lactate was measured in the challenge positive group.

*Diagnostic tests*: Comparing groups and patients with positive and negative challenges, a positive skin test (size and frequency) to raw wheat discriminates patients with oral and inhalant wheat allergy, but not patients with suspected WDEIA [23, 24]. Specific IgE to wheat discriminates the group of challenge positive children and adults with oral wheat allergy from the negative, but with a large overlap between the groups (Figure 4). The level of sIgE to pollen, especially to grass pollen, should be considered a significant confounder as best described by Martens et al. [25]. They challenged a high number of grass pollen patients with elevated levels of sIgE to wheat negative. The level of sIgE to ω-5-gliadin in the group of challenge positive patients with WDEIA is significantly higher than in the negative group, but again with a large overlap and even with negative values in challenge positive patients, as found by Matsuo et al. [26]. Diagnostic precision are thus low for the diagnostic tests, suggesting other allergens may be involved [27]. Measurement of sIgE toward additional wheat related allergens, as described by Pahr et al. [28] and Pastorello et al. [29] might increase the specificity of our sensitization test, but unfortunately serum were not available for retesting.

## Conclusion

Wheat related allergy is a heterogeneous and complex group of diseases, manifesting in different disease entities, rendering a detailed case-history mandatory in each patient. Patient age, occupation and concomitant allergies (food or inhalant) are important factors for evaluation. Diagnostic tests to wheat proteins can be helpful, but a food challenge is required to confirm the diagnosis of a wheat related allergy.

The influence of food matrix, amount and concentration of wheat proteins, the kinetics and underlying mechanisms, as well as a possible role of facilitators need to be addressed further.

## References

[1] Battais F, Richard C, Jacquenet S, Denery-Papini S, Moneret-Vautrin DA (2008). Wheat grain allergies: an update on wheat allergens. Eur Ann Allergy Clin Immunol.

[2] Palosuo K (2003). Update on wheat hypersensitivity. Curr Opin Allergy Clin Immunol.

[3] Morita E, Kunie K, Matsuo H (2007). Food-dependent exercise-induced anaphylaxis. J Dermatol Sci.

[4] Brant A (2007). Baker's asthma. Curr Opin Allergy Clin Immuno.

[5] Moscato G, Vandenplas O, Gerth Van Wijk R, Malo JL, Quirce S, Walusiak J, Castano R, De Groot H, Folletti I, Gautrin D, Yacoub MR, Perfetti L, Siracusa A (2008). Occupational rhinitis. Allergy.

[6] Castano R, Malo JL (2010). Occupational rhinitis and asthma: where do we stand, where do we go?. Curr Allergy Asthma Rep.

[7] Sampson HA (2003). Anaphylaxis and emergency treatment. Pediatrics.

[8] Eller E, Hansen TK, Bindslev-Jensen C (2012). Clinical thresholds to egg, hazelnut, milk and peanut: results from a single-center study using standardized challenges. Ann Allergy Asthma Immunol.

[9] Bruce RA, Blackmon JR, Jones JW, Strait G (1963). Exercising testing in adult normal subjects and cardiac patients. Pediatrics.

[10] Perkins NJ, Schisterman EF (2006). The inconsistency of "optimal" cutpoints obtained using two criteria based on the receiver operating characteristic curve. Am J Epidemiol.

[11] Niggemann B, Sielaff B, Beyer K, Binder C, Wahn U (1999). Outcome of double-blind, placebo-controlled food challenge tests in 107 children with atopic dermatitis. Clin Exp Allergy.

[12] Sicherer SH, Morrow EH, Sampson HA (2000). Dose–response in double-blind, placebo-controlled oral food challenges in children with atopic dermatitis. J Allergy Clin Immunol.

[13] Scibilia J, Pastorello EA, Zisa G, Ottolenghi A, Bindslev-Jensen C, Pravettoni V, Scovena E, Robino A, Ortolani C (2006). Wheat allergy: a double-blind, placebo-controlled study in adults. J Allergy Clin Immunol.

[14] Airaksinen LK, Tuomi TO, Tuppurainen MO, Lauerma AI, Toskala EM (2008). Inhalation challenge test in the diagnosis of occupational rhinitis. Am J Rhinol.

[15] Dohi M, Suko M, Sugiyama H, Yamashita N, Tadokoro K, Juji F, Okudaira H, Sano Y, Ito K, Miyamoto T (1991). Food-dependent, exercise-induced anaphylaxis: a study on 11 Japanese cases. J Allergy Clin Immunol.

[16] Romano A, Di Fonso M, Giuffreda F, Papa G, Artesani MC, Viola M, Venuti A, Palmieri V, Zeppilli P (2001). Food-dependent exercise-induced anaphylaxis: clinical and laboratory findings in 54 subjects. Int Arch Allergy Immunol.

[17] Aihara Y, Takahashi Y, Kotoyori T, Mitsuda T, Ito R, Aihara M, Ikezawa Z, Yokota S (2001). Frequency of food-dependent, exercise-induced anaphylaxis in Japanese junior-high-school students. J Allergy Clin Immunol.

[18] Pacharn P, Jirapongsananuruk O, Daengsuwan T, Vichyanond P, Visitsunthorn N (2009). Wheat-dependent, exercise-induced anaphylaxis in Thai children: a report of 5 cases. Asian Pac J Allergy Immunol.

[19] Keet CA, Matsui EC, Dhillon G, Lenehan P, Paterakis M, Wood RA (2009). The natural history of wheat allergy. Ann Allergy Asthma Immunol.

[20] Eigenmann PA, Sicherer SH, Borkowski TA, Cohen BA, Sampson HA (1998). Prevalence of IgE-mediated food allergy among children with atopic dermatitis. Pediatrics.

[21] Maghni K, Lemière C, Ghezzo H, Yuquan W, Malo J-L (2004). Airway inflammation after cessation of exposure to agents causing occupational asthma. Am J Respir Crit Care Med.

[22] Ring J, Behrendt H, de Weck A (2010). History and classification of anaphylaxis. Chem Immunol Allergy.

[23] Quirce S, Fernandez-Nieto M, Escudero C, Cuesta J, de Las HM, Sastre J (2006). Bronchial responsiveness to bakery-derived allergens is strongly dependent on specific skin sensitivity. Allergy.

[24] Majamaa H, Moisio P, Holm K, Turjanmaa K (1999). Wheat allergy: diagnostic accuracy of skin prick and patch tests and specific IgE. Allergy.

[25] Martens M, Schnoor HJ, Malling HJ, Poulsen LK (2011). Sensitization to cereals and peanut evidenced by skin prick test and specific IgE in food-tolerant, grass pollen allergic patients. Clin Transl Allergy.

[26] Matsuo H, Dahlstrom J, Tanaka A, Kohno K, Takahashi H, Furumura M, Morita E (2008). Sensitivity and specificity of recombinant omega-5 gliadin-specific IgE measurement for the diagnosis of wheat-dependent exercise-induced anaphylaxis. Allergy.

[27] Matsuo H, Kohno K, Niihara H, Morita E (2005). Specific IgE determination to epitope peptides of omega-5 gliadin and high molecular weight glutenin subunit is a useful tool for diagnosis of wheat-dependent exercise-induced anaphylaxis. J Immunol.

[28] Pahr S, Constantin C, Mari A, Scheiblhofer S, Thalhamer J, Ebner C, Vrtala S, Mittermann I, Valenta R (2012). Molecular characterization of wheat allergens specifically recognized by patients suffering from wheat-induced respiratory allergy. Clin Exp Allergy.

[29] Pastorello EA, Farioli L, Conti A, Pravettoni V, Bonomi S, Iametti S, Fortunato D, Scibilia J, Bindslev-Jensen C, Ballmer-Weber B, Robino AM, Ortolani C (2007). Wheat IgE-mediated food allergy in European patients: alpha-amylase inhibitors, lipid transfer proteins and low-molecular-weight glutenins. Allergenic molecules recognized by double-blind, placebo-controlled food challenge. Int arch Allergy Immun.

